# ASXL1 and SETBP1 mutations promote leukaemogenesis by repressing TGFβ pathway genes through histone deacetylation

**DOI:** 10.1038/s41598-018-33881-2

**Published:** 2018-10-26

**Authors:** Makoto Saika, Daichi Inoue, Reina Nagase, Naru Sato, Akiho Tsuchiya, Tomohiro Yabushita, Toshio Kitamura, Susumu Goyama

**Affiliations:** 1 Division of Cellular Therapy, The Institute of Medical Science, The University of Tokyo, 4-6-1 Shirokanedai, Minato-ku, Tokyo 108-8639 Japan; 2 Present Address: Human Oncology and Pathogenesis Program, Department of Medicine, Memorial Sloan Kettering Cancer Center, New York, NY USA

## Abstract

Mutations in *ASXL1* and *SETBP1* genes have been frequently detected and often coexist in myelodysplastic syndrome (MDS) and acute myeloid leukaemia (AML). We previously showed that coexpression of mutant ASXL1 and SETBP1 in hematopoietic progenitor cells induced downregulation of TGFβ pathway genes and promoted the development of MDS/AML in a mouse model of bone marrow transplantation. However, whether the repression of TGFβ pathway in fact contributes to leukaemogenesis remains unclear. Moreover, mechanisms for the repression of TGFβ pathway genes in ASXL1/SETBP1-mutated MDS/AML cells have not been fully understood. In this study, we showed that expression of a constitutively active TGFβ type I receptor (ALK5-TD) inhibited leukaemic proliferation of MDS/AML cells expressing mutant ASXL1/SETBP1. We also found aberrantly reduced acetylation of several lysine residues on histone H3 and H4 around the promoter regions of multiple TGFβ pathway genes. The histone deacetylase (HDAC) inhibitor vorinostat reversed histone acetylation at these promoter regions, and induced transcriptional derepression of the TGFβ pathway genes. Furthermore, vorinostat showed robust growth-inhibitory effect in cells expressing mutant ASXL1, whereas it showed only a marginal effect in normal bone marrow cells. These data indicate that HDAC inhibitors will be promising therapeutic drugs for MDS and AML with *ASXL1* and *SETBP1* mutations.

## Introduction

Mutations in *ASXL1* and *SETBP1* genes have been frequently detected and often coexist in a variety of myeloid neoplasms, including myelodysplastic syndrome (MDS) and acute myeloid leukaemia (AML)^1–3^. *ASXL1* gene is located on chromosome 20q11 and encodes additional sex combs like 1 (ASXL1), which contains a highly conserved ASX homology (ASXH) domain at the N-terminal region and a plant homeodomain (PHD) finger at the C-terminal region^4,5^. ASXL1 interacts with multiple epigenetic regulators, such as EZH2 and BAP1, thereby regulates epigenetic marks and transcription of several target genes, including Hox genes^6,7^. Most *ASXL1* mutations exist in exon 12 of the gene, generating C-terminally truncated mutations. The mutant ASXL1 gains novel functions to form a hyper active complex with BAP1 and to interact with BRD4^8–10^. *SETBP1* gene is located on chromosome 18q21.1 and encodes SET binding protein 1 (SETBP1), which contains a SKI homologous region and a SET-binding region^11^. SETBP1 binds an oncoprotein SET and the resulting heterodimer inhibits a phosphatase PP2A that acts as a tumour suppressor in many cancer cells^12,13^. Mutations of *SETBP1* in the SKI homologous region inhibits its ubiquitination and degradation, resulting in increased expression of SETBP1^14^.

Leukaemic transformation of MDS has had the most impact on the mortality of MDS patients^1,2,15^. A key mechanism of leukaemic transformation of MDS into AML is dysregulation of TGFβ pathway^16,17^. We previously reported that forced expression of a C-terminally truncated ASXL1 mutant in hematopoietic progenitor cells induced MDS-like diseases, and SETBP1 mutations drove leukaemic transformation in ASXL1-mutated MDS in mouse models^18,19^. We also showed global downregulation of TGFβ pathway genes, including *Tgfbr1*, *Tgfbr2*, *Tgfbr3*, *Smad1*, *Smad3*, *Smad4* in cells expressing both ASXL1 and SETBP1 mutations^19^. However, whether the repression of TGFβ pathway in fact contributes to leukaemogenesis induced by ASXL1/SETBP1 mutations remains unclear. Furthermore, mechanisms for the repression of TGFβ pathway genes in ASXL1/SETBP1-mutated MDS/AML cells have not been fully understood.

In this study, we showed that activation of TGFβ pathway indeed inhibits leukaemogenesis induced by ASXL1 and SETBP1 mutations. The repression of TGFβ pathway genes are associated with histone deacetylation at their promoter regions, which can be reversed by treatment with the histone deacetylase (HDAC) inhibitor vorinostat.

## Results

### Activation of TGFβ pathway inhibits leukaemogenesis induced by ASXL1 and SETBP1 mutations

**Figure 1:**
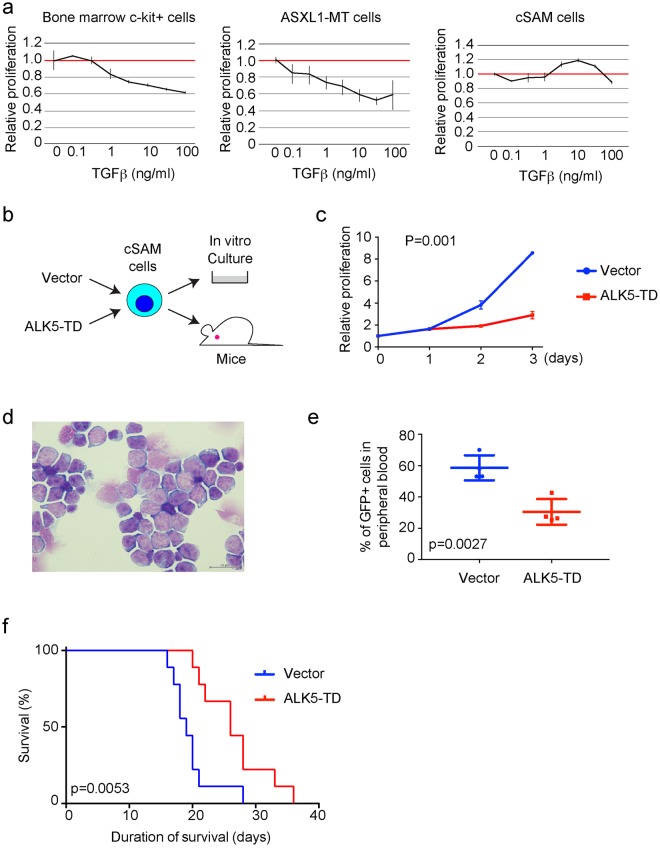
ALK5-TD inhibits leukaemic proliferation of cSAM cells. (**a**) CellTiter-Gro Luminescent Cell Viability Assay using bone marrow c-Kit^+^ cells, ASXL1-MT cells, and cSAM cells, treated with TGFβ at indicated concentrations for 72 h in triplicate. Data are normalized to vehicle control (0 μM group), and are shown as mean ± s.d. (**b**) Experimental scheme used in (**c**–**f**). cSAM cells were transduced with vector or ALK5-TD, and were cultured in the presence of 1 ng/ml IL-3, or were injected (5 × 10^5^ cells/mouse) into recipient mice. (**c**) The growth of 5 × 10^4^/ml cSAM cells transduced with vector or ALK5-TD was estimated using CellTiter-Glo Luminescent Cell Viability Assay from day 1 to day 3. Data are shown as mean ± s.e.m. from triplicated experiments. (**d**) Wright-Giemsa staining of spleen cells collected from moribund mice transplanted with cSAM cells. Scale bar: 50 μm. (**e**) Percentages of leukaemic (GFP^+^) cells in peripheral blood at 16 days post transplantation in mice transplanted with vector- or ALK5-TD-transduced cSAM cells. N = 4 per group. (**f**) Survival curves of the mice transplanted with vector- or ALK5-TD-transduced cSAM cells. N = 9 per group.

We first assessed the role of TGFβ pathway in leukaemogenesis using murine bone marrow cells transformed by a C-terminally truncated form of ASXL1 mutant [ASXL1-MT cells: cells expressing ASXL1 mutation (ASXL1-MT)]^18^ or those transformed by combined expression of SETBP1-D868N and ASXL1-MT (cSAM cells: cells with combined expression of SETBP1 and ASXL1 Mutations)^19^. SETBP1-D868N is an oncogenic mutation of SETBP1, and ASXL1-MT is a leukaemia-associated ASXL1 mutant [ASXL1 (1900–1922del; E635RfsX15)]. In a previous study, we showed that TGFβ pathway genes were specifically downregulated in cSAM cells but not in ASXL1-MT cells^19^. Consistent with this observation, TGFβ inhibited the growth of normal bone marrow c-Kit^+^ cells and ASXL1-MT cells in a dose-dependent manner, whereas it showed little effect on the growth of cSAM cells (Fig. 1a). Thus, cSAM cells that express low level of TGFβ pathway genes are poorly responsive to TGFβ-induced growth suppression. To assess the effect of forced activation of TGFβ pathway in cSAM cells, we next transduced vector or ALK5-TD (a constitutively active form of Tgfb receptor 1) into cSAM cells, and cultured the cells *in vitro* or directly transplanted them into recipient mice (Fig. 1b). ALK5-TD-transduced cSAM cells grew slower than vector-transduced cells *in vitro* (Fig. 1c). Furthermore, we observed impaired engraftment and significantly delayed leukaemia development in mice receiving ALK5-TD-transduced cSAM cells relative to those receiving control cells (Fig. 1d–f). These data indicate that repression of TGFβ pathway contributes to leukaemic proliferation of cSAM cells both *in vitro* and *in vivo*.

### Vorinostat promotes transcription of TGFβ pathway genes in cSAM cells

**Figure 2:**
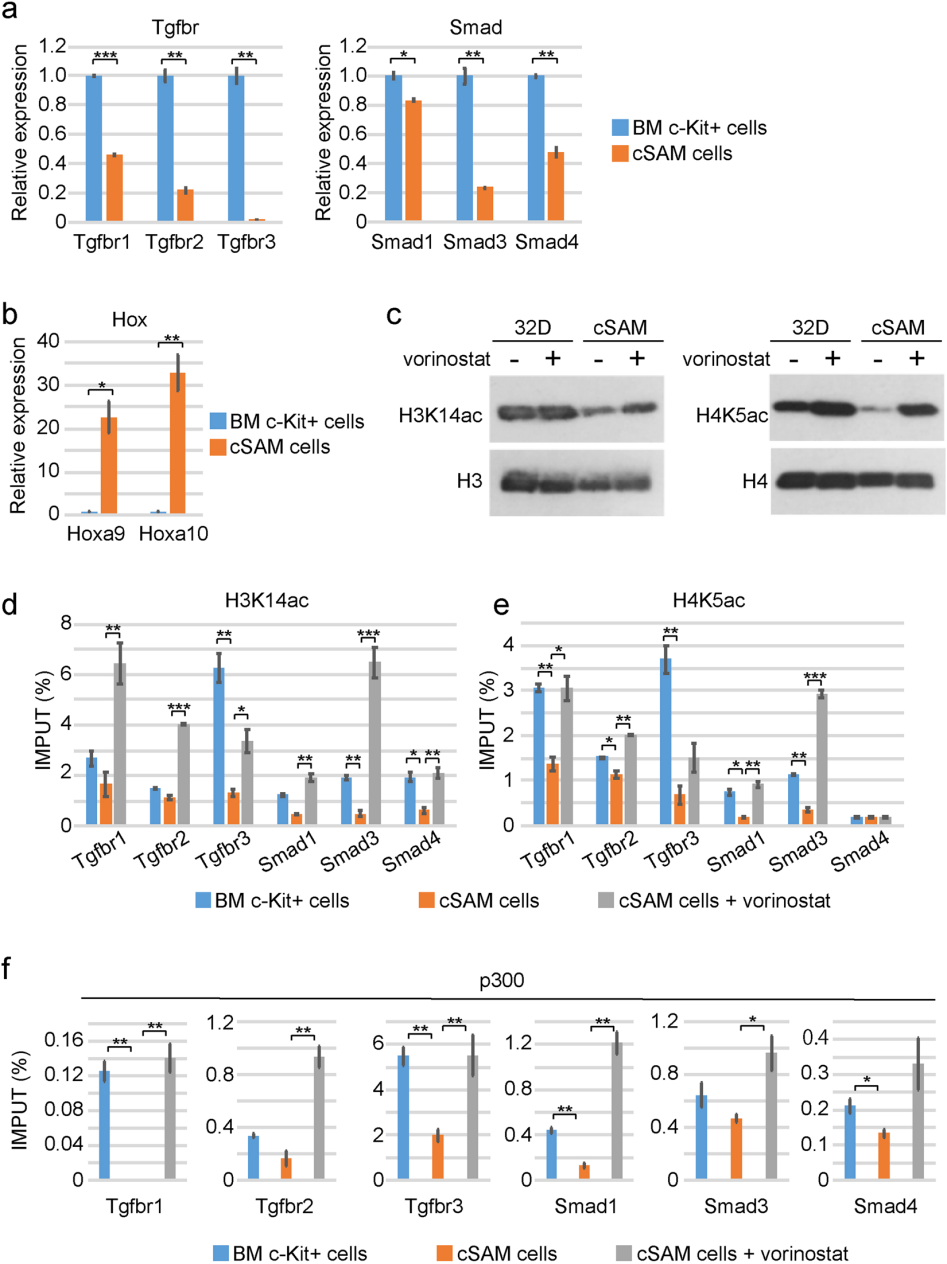
Vorinostat increases acetylation of lysine residues on histone H3 and H4 in cSAM cells. (**a**,**b**) Expression of TGFβ pathway genes was decreased (**a**), while expression of *Hoxa9* and *Hoxa10* was increased (**b**) in cSAM cells compared to normal bone marrow c-Kit+ cells. Data are shown as mean ± s.e.m. from duplicate experiments. *P < 0.05, **P < 0.01, ***P < 0.001, Student’s t-test. (**c**) Western blotting for histone acetylation in 32D cells and cSAM cells. Vorinostat increased both H3K14ac and H4K5ac in cSAM cells. Full-length blots are shown in Supplemental Fig. 2. (**d**–**f**) Genomic DNA fragments from control [normal bone marrow (BM) c-kit + cells] and cSAM cells cultured with 1 µM vorinostat or vehicle control (DMSO) were immunoprecipitated with anti-H3K14ac (**d**), anti-H4K5ac (**e**) and anti-p300 (**f**) antibodies. Enrichments of H3K14ac and H4K5ac at transcription starting sites (TSS) of *Tgfbr1*, *Tgrbr2*, *Tgfbr3*, *Smad1*, *Smad3*, *and Smad4* were measured by qPCR. Data are shown as mean ± s.e.m. *P < 0.05, **P < 0.01, ***P < 0.001, Student’s t-test.

**Figure 3:**
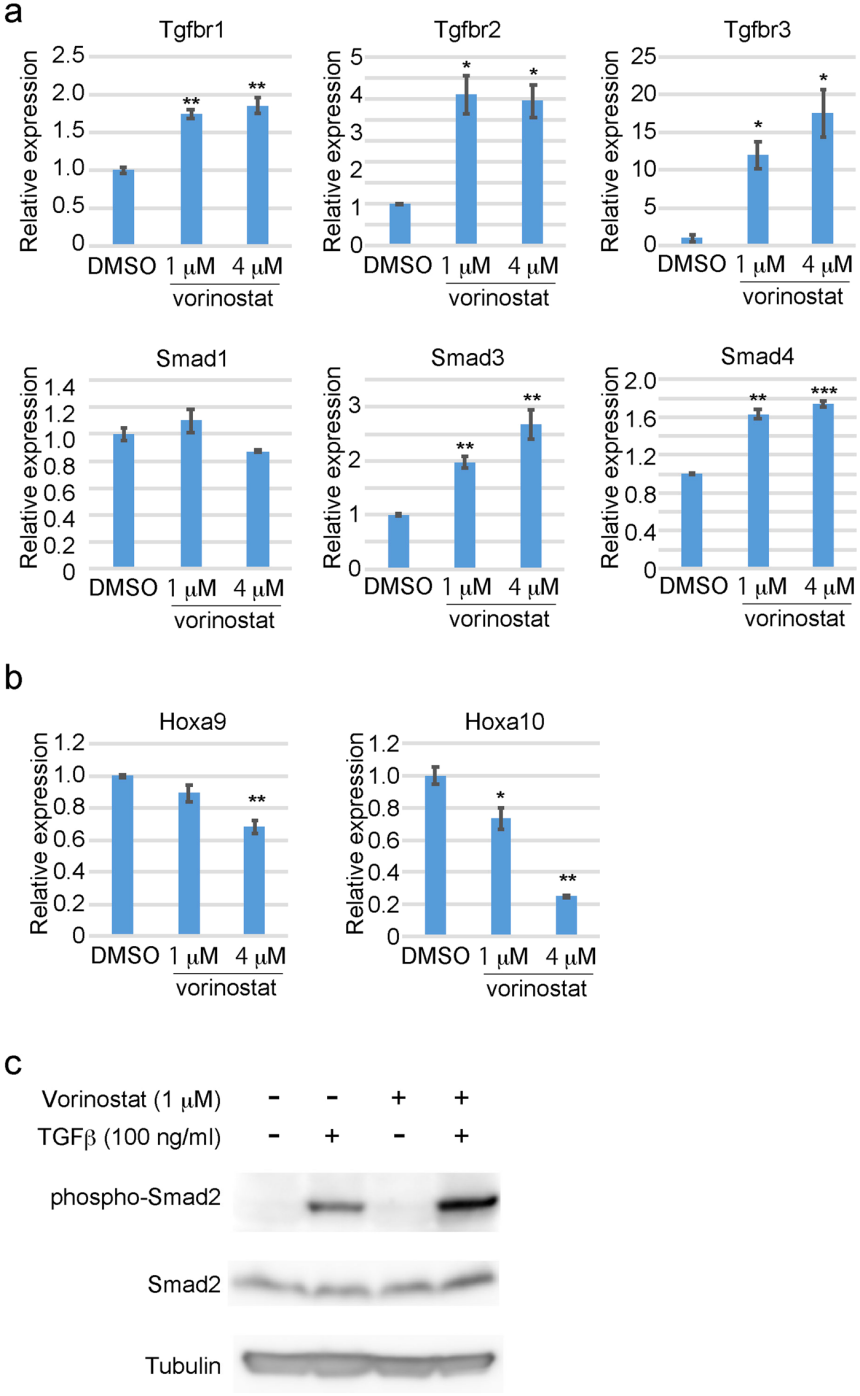
Vorinostat upregulated TGFβ pathway genes and activates TGFβ pathway in cSAM cells. (**a**,**b**) cSAM cells were cultured with 1 or 4 µM vorinostat or vehicle control (DMSO) for 6 hours, and expression of TGFβ pathway genes (**a**) and Hox genes (**b**) was analyzed by qPCR. Results were normalized to *GAPDH*, with the relative mRNA level in vehicle-treated cells set at 1. Data are shown as mean ± s.e.m. of duplicate wells. *P < 0.05, **P < 0.01, ***P < 0.001, Student’s t-test. (**c**) cSAM cells were treated with vorinostat (1 μM) for 24 hours, following TGFβ (100 ng/ml) stimulation for 45 minutes. TGFβ-induced phosphorylation of Smad2 was substantially increased in vorinostat-treated cSAM cells.

Histone acetylation is associated with active transcription, and it was recently shown that SETBP1 represses Runx1 expression through histone deacetylation^20^. We therefore examined levels of histone acetylation around the promoter regions of TGFβ pathway genes in c-Kit^+^ cSAM cells and normal c-Kit^+^ bone marrow progenitor cells. Consistent with our previous report^19^, multiple TGFβ pathway genes (*Tgfbr1*, *Tgfbr2*, *Tgfbr3*, *Smad1*, *Smad3*, and *Smad4*) were downregulated, whereas Hox genes (*Hoxa9* and *Hoxa10*) were upregulated in cSAM cells compared with control cells (Fig. 2a,b). Chromatin immunoprecipitation coupled with quantitative PCR (ChIP-qPCR) revealed the decreased acetylation of several histone lysine residues, especially of histone H3 lysine 14 (H3K14) and histone H4 lysine 5 (H4K5), at the vicinity of transcription starting sites (TSSs) of multiple TGFβ pathway genes in cSAM cells. Treatment of cSAM cells with vorinostat resulted in enhanced acetylation of H3K14 and H4K5, particularly at the promotor regions of TGFβ pathway genes (Fig. 2c–e, Supplemental Fig. 1). We also observed decreased binding of a histone acetyltransferase p300 at the same promoter regions, which was reversed by vorinostat (Fig. 2f). Vorinostat treatment also induced upregulation of *Tgfbr1*, *Tgfbr2*, *Tgfbr3*, *Smad3* and *Smad4* in cSAM cells (Fig. 3a). In contrast, expression of *Hoxa9* and *Hoxa10* were not upregulated, but were rather downregulated by vorinostat in cSAM cells (Fig. 3b). We then assessed responsiveness of vehicle- or vorinostat-treated cSAM cells to TGFβ. As expected, TGFβ-induced phosphorylation of Smad2, a key intracellular mediator of TGFβ signaling, was substantially increased in vorinostat-treated cSAM cells (Fig. 3c). These results indicate that repression of TGFβ pathway genes are mainly caused by histone deacetylation, which can be reversed by the HDAC inhibitor vorinostat.

### Vorinostat inhibits leukaemogenesis induced by ASXL1 and SETBP1 mutations

**Figure 4:**
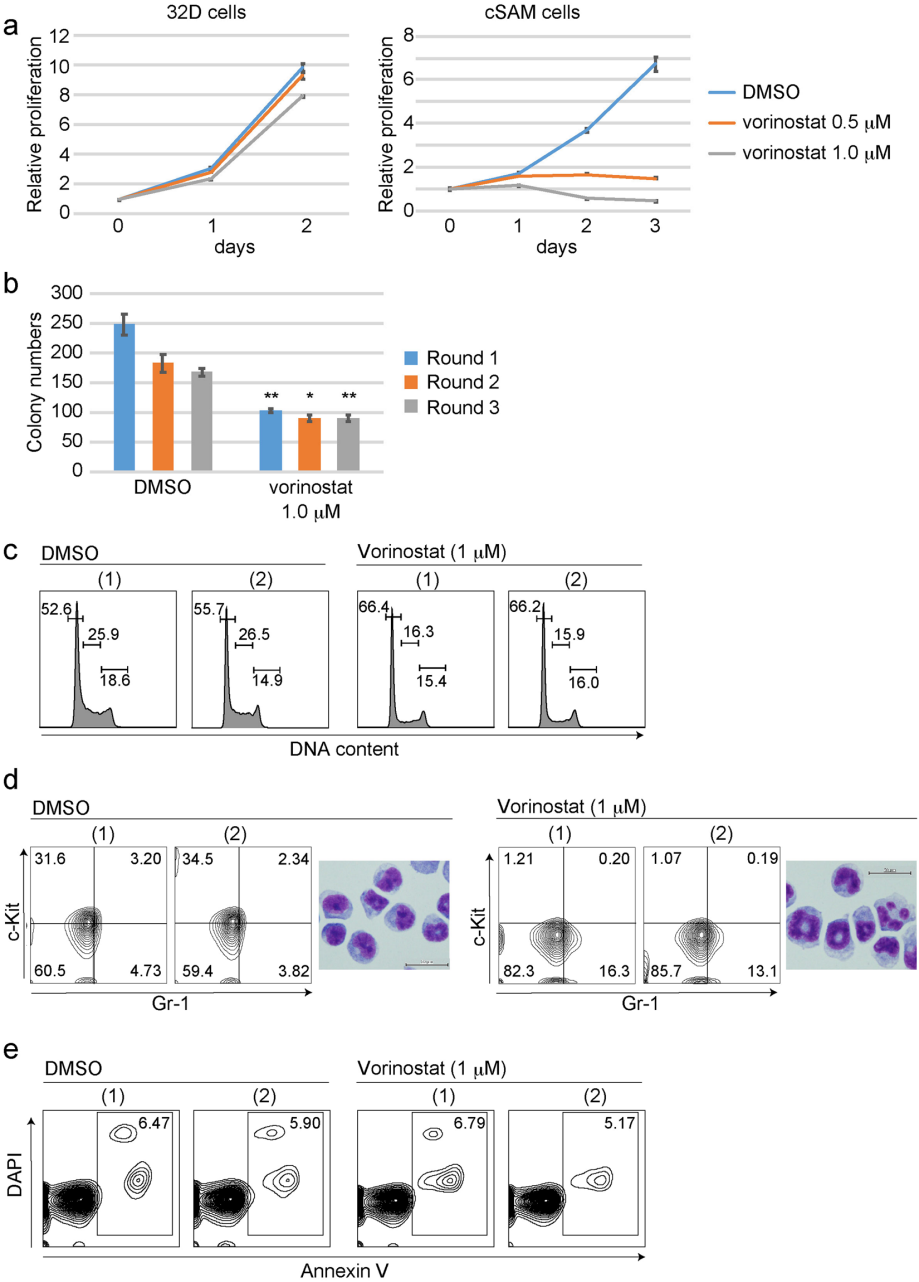
Vorinostat inhibits leukaemic proliferation of cSAM cells. (**a**) cSAM cells and 32D cells were cultured in the presence of 1 ng/ml IL-3 together with 0.5 or 1 μM vorinostat or vehicle control (DMSO). The growth of the cells was estimated using CellTiter-Glo Luminescent Cell Viability Assay. All data with error bars indicate the mean ± s.e.m. from triplicated experiments. (**b**) c-kit^+^ cSAM cells were serially replated in methylcellulose supplemented with SCF (100 ng/ml), IL-3 (10 ng/ml), together with vorinostat (0.1 μM) or vehicle control (DMSO). Shown are colony counts per 3 × 10^2^ replated cells (mean ± s.e.m) from duplicate plates at 1^st^, 2^nd^ and 3^rd^ rounds. *P < 0.05, **P < 0.01, Student’s t-test. (**c**–**e**) Cell-cycle status, differentiation and apoptosis were assessed after 6 hours culture with vorinostat (1 μM). (**c**) FACS profiles of vehicle- or vorinostat-treated cSAM cells are shown. The numbers indicate the percentages of cells in the G0/G1, S and G2/M phases. (**d**) Shown are FACS profiles of Gr-1 and c-Kit expression, and Wright-Giemsa staining of cytospin preparations of vehicle- or vorinostat-treated cSAM cells. The numbers indicate the percentages of cells in each gate. Scale bars; 50 μm. (**e**) Shown are FACS profiles of Annexin V and DAPI expression of vehicle- or vorinostat-treated cSAM cells. The numbers indicate the percentages of Annexin V+ cells.

Next, we examined the effect of vorinostat on the growth of cSAM cells. Addition of low-dose vorinostat (0.5 μM) was sufficient to inhibit proliferation of cSAM cells in culture, whereas even high dose vorinostat (1 μM) only showed a marginal effect on the growth of a mouse myeloid cell line, 32D (Fig. 4a). We then performed colony replating assay using cSAM cells in the presence or absence of vorinostat. Again, addition of vorinostat (0.1 μM) reduced colony forming capability of cSAM cells (Fig. 4b). To characterize the decreased cell growth of cSAM cells induced by vorinostat, we analyzed cell cycle status, differentiation, and apoptosis in vehicle- or vorinostat-treated cSAM cells. Vorinostat treatment consistently decreased the proportion of S phase cells (Fig. 4c) and the frequency of immature cells (c-Kit^+^Gr-1^−^ cells) in culture (Fig. 4d). In contrast, vorinostat did not induce apoptosis of cSAM cells (Fig. 4e). Thus, vorinostat inhibits the growth of cSAM cells mainly by inducing cell cycle arrest and differentiation.

**Figure 5:**
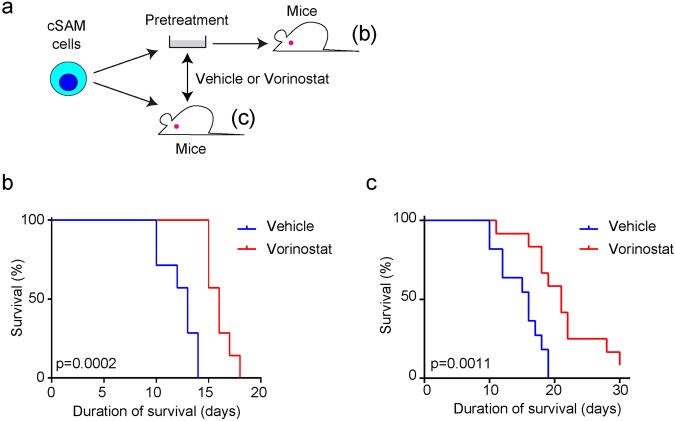
Vorinostat inhibits the development of cSAM cell-driven MDS/AML *in vivo*. (**a**) Experimental scheme used in (**b**,**c**). cSAM cells were pretreated with 1 mM vorinostat or vehicle control (DMSO) for 6 hours before transplantation, or directly injected into recipient mice following oral administration of vorinostat (50 mg/kg) to the recipient mice. (**b**) Overall survival of mice transplanted with vehicle- or vorinostat-pretreated cSAM cells. N = 7 per group. (**c**) Overall survival of mice that were orally treated with vehicle or vorinostat after transplantation. N = 11 (vehicle) or 12 (vorinostat). P value was calculated using a log-rank test.

To assess the effect of vorinostat on the development of cSAM cell-driven MDS/AML *in vivo*, we performed two independent assays (Fig. 5a). First, we pretreated cSAM cells with 1 μM vorinostat or vehicle control for 6 hours and transplanted these cells into recipient mice. As shown in Fig. 5b, pretreatment of cSAM cells with vorinostat significantly delayed the development of leukaemia *in vivo*. Second, we directly transplanted cSAM cells into recipient mice, and the mice were treated with vehicle or vorinostat from day 2 to day 9. This *in vivo* treatment with vorinostat also prolonged survival of the recipient mice transplanted with cSAM cells (Fig. 5c). Thus, vorinostat has the robust *in vivo* effect to inhibit leukaemogenesis induced by ASXL1 and SETBP1 mutations.

### Vorinostat inhibits the growth of other MDS/AML cells expressing mutant ASXL1

**Figure 6:**
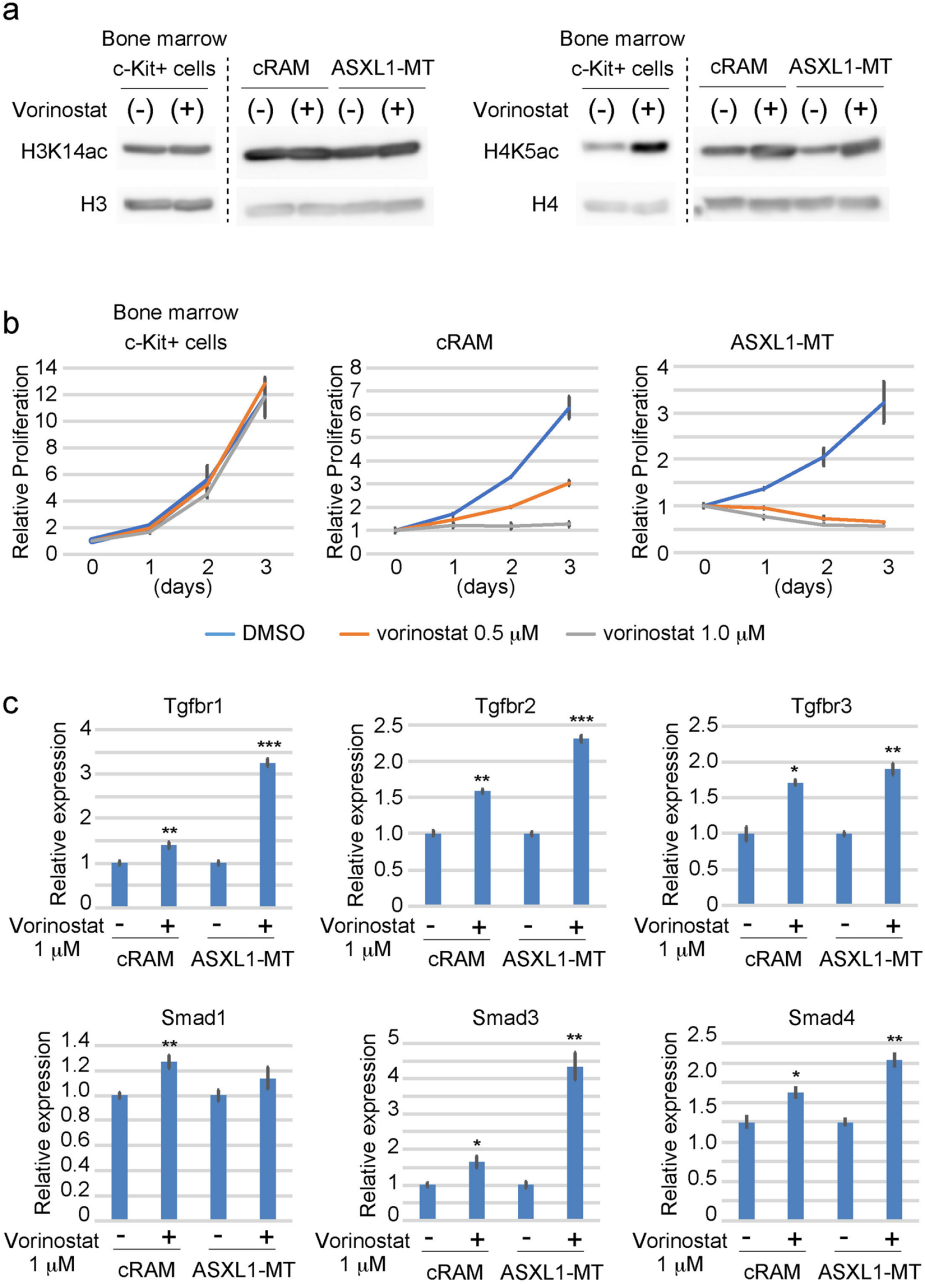
Vorinostat inhibits the growth of other MDS/AML cells expressing ASXL1-MT. (**a**) Western blotting for histone acetylation in bone marrow c-Kit^+^ cells, cRAM cells and ASXL1-MT cells. Full-length blots are shown in Fig. S3. Vorinostat increased H4K5ac in all these cells. (**b**) Bone marrow c-Kit^+^ cells, cRAM cells and ASXL1-MT cells were cultured in the presence of 1 or 10 ng/ml IL-3 together with 0.5 or 1 μM vorinostat or vehicle control (DMSO). The growth of the cells was estimated using Cell Counting Kit-8. All data with error bars indicate the mean ± s.e.m. from triplicated experiments. (**c**) cRAM cells and ASXL1-MT cells were cultured with 1 µM vorinostat or vehicle control (DMSO) for 6 hours, and expression of TGFβ pathway genes was analyzed by qPCR. Results were normalized to *GAPDH*, with the relative mRNA level in vehicle-treated cells set at 1. Data are shown as mean ± s.e.m. of duplicate wells. *P < 0.05, **P < 0.01, ***P < 0.001, Student’s t-test.

Finally, we assessed the effect of vorinostat on other murine MDS/AML cells expressing mutant ASXL1; ASXL1-MT cells and cRAM cells. cRAM (combined expression of RUNX1 and ASXL1 Mutations) cells were generated by transducing a C-terminally truncated RUNX1 mutant (RUNX1-S291fsX300^21^) into bone marrow progenitors derived from the recently established conditional knock-in mice expressing ASXL1-MT^22^. We cultured these MDS/AML cells and bone marrow c-Kit^+^ cells in the presence/absence of vorinostat. Addition of vorinostat in culture resulted in increased acetylation of H4K5 in all these cells (Fig. 6a) and effectively inhibited the growth of cRAM cells and ASXL1-MT cells (Fig. 6b). In contrast, bone marrow c-Kit^+^ cells grew normally in the presence of vorinostat (Fig. 6b). Vorinostat also induced upregulation of *Tgfbr1*, *Tgfbr2*, *Tgfbr3*, *Smad1*, *Smad3*, and *Smad4* in both cRAM and ASXL1-MT cells (Fig. 6c). These data suggest that vorinostat upregulates TGFβ pathway genes and inhibits cell growth in a wide range of MDS/AML cells.

## Discussion

Clinical data revealed frequent coexistence of ASXL1 and SETBP1 mutations in myeloid neoplasms, and we previously demonstrated their cooperation to promote leukaemic transformation in mouse MDS/AML models^19^. We extended the research, and showed that activation of TGFβ pathway inhibits leukaemogenesis induced by both mutations. Aberrant TGFβ signaling has been implicated in a variety of haematopoietic neoplasms. Loss of Smad3 promotes the development of T-cell lineage acute lymphoblastic leukaemia in concert with loss of p27(kip1)^23^. Several leukaemia-associated oncogenes, EVI1, RUNX1-EVI1 and RUNX1-ETO, interact with Smad3 to repress TGFβ signaling in AML and chronic myeloid leukaemia^23–25^. Mutations in *Smad4* gene that disrupt its transcriptional activity has been detected in AML^26^. Loss of TGFBRI and TGFBRII expression was shown to trigger the development of myeloid or lymphocytic leukaemia^27^. Here we identified epigenetic repression of multiple TGFβ pathway genes as another mechanism to promote leukaemogenesis.

Recently, Vishwakarma *et al*. reported that SETBP1 recruits a nucleosome remodeling deacetylase (NuRD) complex containing HDAC1 to Runx1 promoters, thereby represses its expression. They also showed that HDAC inhibitors restore Runx1 expression and inhibit leukaemogenesis driven by SETBP1 overexpression^20^. These findings, together with our data, indicate that SETBP1 plays a key role in inducing aberrant histone acetylation around promoter regions of several tumor suppressor genes. However, vorinostat treatment also induced upregulation of TGFβ pathway genes in MDS/AML cells that express only the ASXL1 mutant (ASXL1-MT cells and cRAM cells). In addition, our previous Liquid Chromatography-Mass Spectrometry analysis revealed physical interaction between mutant ASXL1 and HDAC1^10^. These observations suggest the possible involvement of ASXL1 mutations in the regulation of histone acetylation. How the complexes containing ASXL1, SETBP1 and HDAC1 select their target genes warrants further investigation.

Interestingly, vorinostat treatment resulted in significant downregulation of *Hoxa9* and *Hoxa10* in cSAM cells. Given that both ASXL1 and SETBP1 mutations were shown to upregulate Hox gene expression^19,28^, vorinostat may directly modulate activities of ASXL1 and/or SETBP1 mutants through histone acetylation-independent functions. Such effects of HDAC inhibitors have been repeatedly shown for a variety of non-histone proteins^29–31^. The potential role of acetylation to regulate activities and stabilities of ASXL1 and SETBP1 proteins will merit future studies.

Importantly, neither ALK5-TG overexpression nor vorinostat treatment completely inhibited the development of MDS/AML induced by cSAM cells. We also found that vorinostat did not induce apoptosis, whereas it effectively induced cell cycle arrest and differentiation in cSAM cells. The lack of apoptosis-inducing effect of vorinostat on cSAM cells may account for its limited therapeutic efficacy. Combined therapy with vorinostat and inhibitors of anti-apoptosis proteins, such as venetoclax^32^, could show synergistic effects against MDS/AML cells.

In summary, we showed decreased histone acetylation around promoters of TGFβ pathway genes in MDS/AML cells expressing mutant ASXL1 and SETBP1. The HDAC inhibitor vorinostat increased levels of histone acetylation, reversed transcription of the TGFβ pathway genes, and inhibited leukaemic proliferation of ASXL1/SETBP1-mutated cells. Vorinostat also inhibited the growth of other MDS/AML cells expressing ASXL1-MT. These findings suggest that HDAC inhibitors will be promising therapeutic drugs to treat myeloid neoplasms with *ASXL1* and/or *SETBP1* mutations.

## Materials and Methods

### Mice

C57BL/6 (Ly5.1) mice (Sankyo Labo Service Corporation, Tokyo, Japan) and C57BL/6 (Ly5.2) mice (Charles River Laboratories Japan, Yokohama, Japan) were used for bone marrow transplantation assays. All animal studies were approved by the Animal Care Committee of the Institute of Medical Science at the University of Tokyo (approval number: PA13-19, PA16-31, PA17-75), and were conducted in accordance with the Regulation on Animal Experimentation at University of Tokyo based on International Guiding Principles for Biomedical Research Involving Animals.

### Plasmids

The plasmid pcDNA3-HASL-ALK5(TD) was provided by Dr. Miyazono K. (Tokyo university, Japan), and we cloned it into the vector pMYs-IRES-Puromicin-resistant gene (pMYs-IP).

### Retrovirus and transduction

As described previously^21^, retroviruses were generated by transient transfection of Plat-E packaging cells with the calcium-phosphate coprecipitation method.

### Establishment of murine MDS/AML cells

To generate cSAM cells, we transduced ASXL1-MT and SETBP-D868N into mouse bone marrow progenitor cells, and transplanted these cells into sublethally irradiated recipient mice. Leukaemic cells were isolated from bone marrow of the moribund mice, and their leukaemogenic activity was confirmed by serial transplantations^19^. ASXL-MT cells were established through serial passages in mice using mouse bone marrow progenitor cells transduced with ASXL1-MT alone^18^. To generate cRAM cells, we transduced RUNX1-S291fsX300^21^ into mouse bone marrow progenitor cells derived from the recently established conditional knock-in mice expressing ASXL1-MT^22^. The cells expressing RUNX1 and ASXL1 mutations were transplanted into sublethally irradiated recipient mice to establish mouse MDS/AML cells.

### Cell culture

cSAM cells, ASXL1-MT cells, cRAM cells and 32D cells were cultured in RPMI-1640 medium supplemented with 10% fetal bovine serum and 1 ng/ml interleukin-3 (IL-3) (R&D Systems, catalog #403-ML). Mouse bone marrow c-Kit+ cells were cultured in RPMI-1640 medium supplemented with 10% fetal bovine serum and 10 ng/ml interleukin-3 (IL-3) (R&D Systems, catalog #403-ML) . In some experiments, we added the indicate concentrations of TGFβ (R&D Systems, catalog #70-MB) in the culture. The growth of viable cells in culture was measured using the CellTiter-Gro Luminescent Cell Viability Assay (Promega, Madison, WI, USA) or the Cell Counting Kit-8 (Dojindo, Kumamoto, Japan).

### Colony-forming assay

c-Kit^+^ cells were isolated from mouse bone marrow cells and cSAM cells using MACS (magnetic-activated cell sorting), and were cultured in Methocult 3231 (StemCell Technologies, Vancouver, BC, Canada) supplemented with 20 ng/ml SCF and 10 ng/ml mouse IL-3. A total of 300 cells were plated in duplicates for each round of plating. Colony counting and replating were performed every 5 days.

### Transplantation assay

cSAM cells were infected for 72 hours with the retroviruses harbouring pMYs-IP and pMYs-IP-ALK5-TD, using 6-well dishes coated with RetroNectin (Takara Bio, Otsu, Japan). The infected cells were selected using 1 μg/ml puromycin, and 5 × 10^5^ infected cells were transplanted intravenously into non-radiated recipient mice. For experiments using vorinostat, cSAM cells were cultured in RPMI-1640 medium supplemented with 1 ng/ml IL-3 and 1 μM vorinostat or DMSO for 6 hours. These precultured cells (2 × 10^5^/mouse) were transplanted into recipient mice. In some experiments, cSAM cells were directly transplanted into recipient mice (2 × 10^5^/mouse), and vehicle control or vorinostat (50 mg/kg/day) were orally administered to these mice from day 2 to day 9. Overall survival of transplanted mice was calculated using the Kaplan-Meier method.

### Western blot analysis

For analyzing histone modifications, cells were cultured with 1 µM vorinostat or DMSO for 6 hours, and were lysed in IP buffer (150 mM NaCl, 50 mM Tris pH 7.5, 1 mM EDTA, 1% Triton, 2 mM sodium orthovanadate, 2 mM PMSF, 50 mM sodium fluoride)^18^. Pelleted nuclei were resuspended with 0.2 M sulfuric acid, and histones were precipitated from the supernatant with trichloroacetic acid (TCA). In other experiments, cells were cultured with 1 µM vorinostat or DMSO for 24 hours, or with 100 ng/ml TGFβ (R&D Systems, catalog #70-MB) for 45 minutes, and were lysed directly in 1x Laemmli Sample Buffer. Whole-cell lysates were subjected to sodium dodecyl sulfate–polyacrylamide gel electrophoresis and transferred to a polyvinylidene fluoride membrane (Bio-Rad). The blot was incubated with the anti-histone antibodies, anti-acetylated histone antibodies, anti-Smad2/3 (#3102; Cell Signaling Technology, Beverly, MA), anti-phospho-Smad2 (Ser465/Ser467, clone E8F3R, #18338, Cell Signaling Technology, Beverly, MA) and anti-Tubulin (clone B-5-1-2; Santa Cruz Biotechnology Santa Cruz, CA). Signals were detected with SuperSignalWest Pico (Pierce, Thermo Fisher Scientific) and visualized on films or with imagequant LAS 4000 (Fujifilm Life Science, Roche Diagnostics). All antibodies for histones (H3, H4, H3K14ac, H4K5ac, H4K8ac, H4K16ac) were kind gifts from Dr. Kimura H. (Tokyo Institute of Technology, Japan)^33^.

### Flow cytometry

Cells were stained by fluoro-conjugated antibodies [anti-mouse CD117 (BioLegend, catalog #105808, clone 2B8, 1:400), and anti-mouse Gr-1 (BioLegend, catalog #108411, clone RB6-8C5, 1:400)] for 30 min at 4 °C. After staining, cells were washed with cold PBS several times, and were resuspended with PBS containing 2% FBS. Cells were analyzed on a FACS Verse (BD Biosciences, San Jose, CA, USA). Cell cycle analysis (Cycletestt^m^ Plus DNA Reagent Kit, BD Biosciences) and apoptosis analysis (Annexin V-APC kit; BD Biosciences) were performed according to the manufacturer’s recommendations. The data were analyzed using FlowJo software (Treestar, Inc., San Carlos, CA).

### Quantitative PCR

As described previously^33^, total RNA was extracted from BM cells using the RNeasy Mini kit (QIAGEN), and reverse-transcribed using the High Capacity cDNA Reverse Transcription Kit (Applied Biosystems, Foster City, CA, USA) with the deoxyribonuclease I (Invitrogen - Thermo Fisher Scientific - MA, USA). Quantitative PCR (qPCR) was performed using SYBR Premix EX Taq (Takara Bio) and Rotor-Gene Q (Qiagen, Venlo, The Netherlands). Sequences of the primers used for qPCR in this study, from 5′ to 3′ are as follows:

Tgfbr1 Fw: TCTGCATTGCACTTATGCTGA, Tgfbr1 Rv: AAAGGGCGATCTAGTGATGGA, Tgfbr2 Fw: CCGCTGCATATCGTCCTGTG, Tgfbr2 Rv: AGTGGATGGATGGTCCTATTACA, Tgfbr3 Fw: GGTGTGAACTGTCACCGATCA, Tgfbr3 Rv: GTTTAGGATGTGAACCTCCCTTG, Smad1 Fw: GCTTCGTGAAGGGTTGGGG, Smad1 Rv: CGGATGAAATAGGATTGTGGGG, Smad3 Fw: CACGCAGAACGTGAACACC, Smad3 Rv: GGCAGTAGATAACGTGAGGGA, Smad4 Fw: ACACCAACAAGTAACGATGCC, Smad4 Rv: GCAAAGGTTTCACTTTCCCCA.

### Chromatin Immunoprecipitation assay (ChIP)

cSAM cells were cultured with 1 µM vorinostat or vehicle control (DMSO) for 6 hours. Genomic DNA fragments from these c-kit^+^ cSAM cells and normal c-kit^+^ bone marrow progenitor cells were immunoprecipitated using anti-acetylated-histone (same antibodies used for western blotting) and anti-p300 antibodies (Santa Cruz 584) as described previously^34^. Quantitative PCR was performed with a Rotor-Gene Q (Qiagen) using SYBR Premix EX Taq (Takara). Sequences of the primers used for ChIP-qPCR in this study, from 5′ to 3′ are as follows:

Tgfbr1 Fw: CAGTTACAAAGGGCCGGAGC, Tgfbr1 Rv: CAACACGATGAGGAGCTGCG, Tgfbr2 Fw: TGTAGAGTCCAGGCAAGGCT, Tgfbr2 Rv: GACTCACTCATCAACTTTACCCG, Tgfbr3 Fw: CCCTTTCGCTGATTGCTGTG, Tgfbr3 Rv: CTTCTCCCTCTCCACCTCTCT, Smad1 Fw: GGGACTAGAGTCAGAGGAAGG, Smad1 Rv: CGAGCCTGGATTGATCTGGT, Smad3 Fw: CAGAGGAGGAGGAGGAGGAG, Smad3 Rv: GTGGCAGTAGAAAGTTTGGGTT, Smad4 Fw: CTCGCTCGCTGCTCAAACTC, Smad4 Rv: GGTAATTTCAGGGTTTGGCCC.

### Morphological analysis

Cytospin preparations were stained with Giemsa. Images were obtained with a BX51 microscope and a DP12 camera (Olympus).

### Statistics

Statistical significance was determined by the indicated tests for independent variables using GraphPad Prism 7 (GraphPad Software Inc., La Jolla, CA). Statistical analyses for evaluating differences between two groups were performed by the unpaired and two-tailed Student’s t test. The survival distributions were compared by the log-rank test. No specific statistical methods were used to predetermine the sample size.

## Electronic supplementary material

Supplemental Figures

## Data Availability

All data generated or analyzed during this study are included in this published article. The datasets generated during the current study are available from the corresponding author on reasonable request.
