# Supplementary material for: ASXL1 and SETBP1 mutations promote leukaemogenesis by repressing TGFβ pathway genes through histone deacetylation
Source: Sci Rep. 2018 Oct 26;8:15873. doi: 10.1038/s41598-018-33881-2 (PMC6203835; doi:10.1038/s41598-018-33881-2)

Supplemental Information

ASXL1 and SETBP1 mutations promote leukaemogenesis by repressing TGFβ pathway genes through histone deacetylation

Makoto Saika[1], Daichi Inoue[1,2], Reina Nagase[1], Naru Sato[1], Akiho Tsuchiya[1], Tomohiro Yabushita[1], Toshio Kitamura[1*], Susumu Goyama[1*]

[1]Division of Cellular Therapy, The Institute of Medical Science, The University of Tokyo, 4-6-1 Shirokanedai, Minato-ku, Tokyo 108-8639, Japan

[2](current address) Human Oncology and Pathogenesis Program, Department of Medicine, Memorial Sloan Kettering Cancer Center, New York, NY, USA.

Supplemental Figure 1

Supplemental Figure 2

Supplemental Figure 3

Supplemental Figure 1

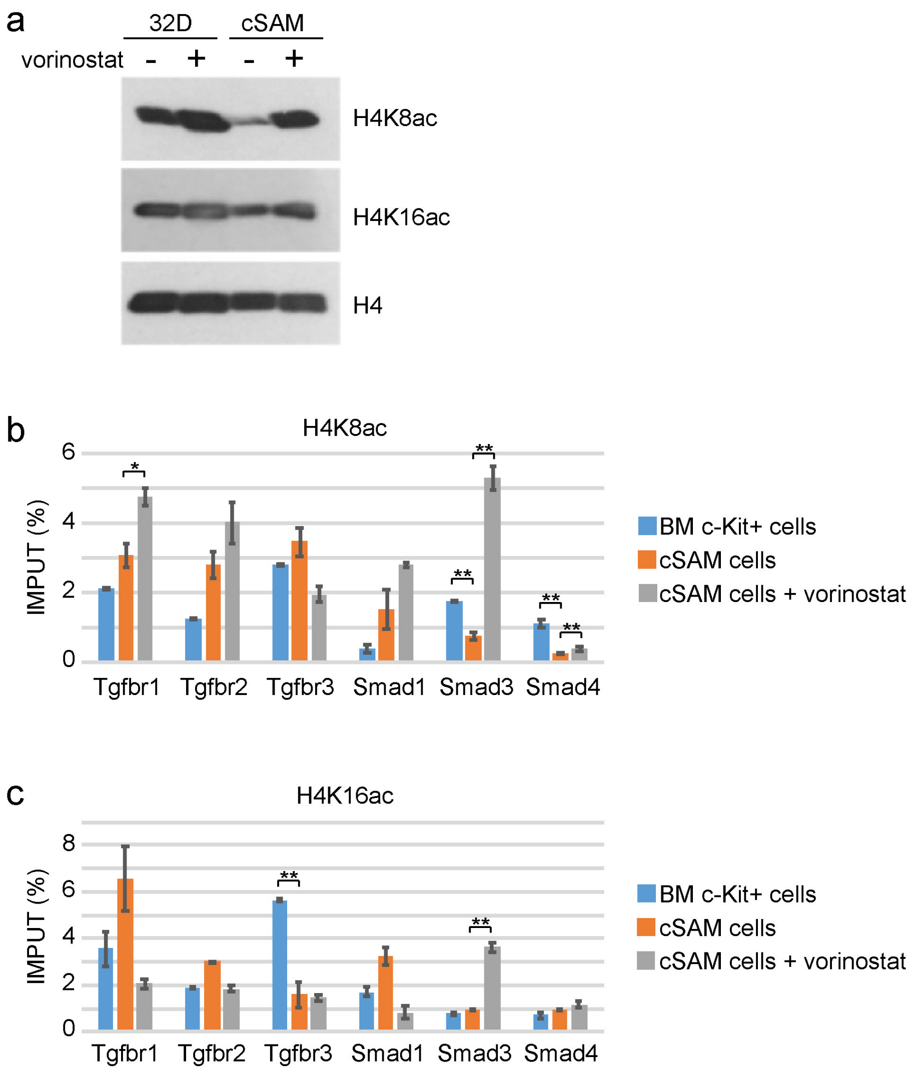

Supplemental Figure 1.

(**a**) Western blotting for histone acetylation in 32D cells and cSAM cells. Vorinostat increased H3K8ac and H4K16ac in cSAM cells. Full-length blots are shown in Supplemental Figure 2.

(**b, c**) Genomic DNA fragments from bone marrow c-kit+ cells and cSAM cells cultured with 1 μM vorinostat or vehicle control (DMSO) were immunoprecipitated with anti-H4K8ac (**b**) and anti-H4K16ac (**c**) antibodies. Enrichments of H3K14ac and H4K16ac at transcription starting sites of Tgfbr1, Tgrbr2, Tgfbr3, Smad1, Smad3, and Smad4 were measured by qPCR. Data are shown as mean ± s.e.m.

*P<0.05, **P<0.01, Student t-test.

Supplemental Figure 2

Full blots for Figure 2b & Supplemental Figure 1

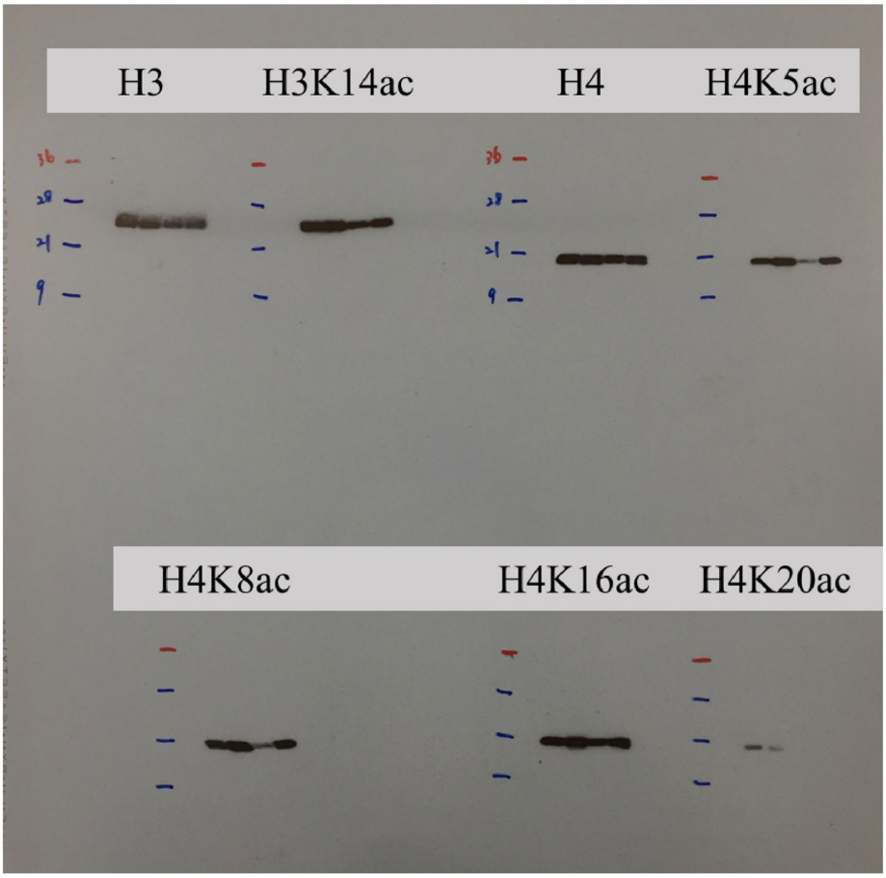

Full blots for Figure 3c

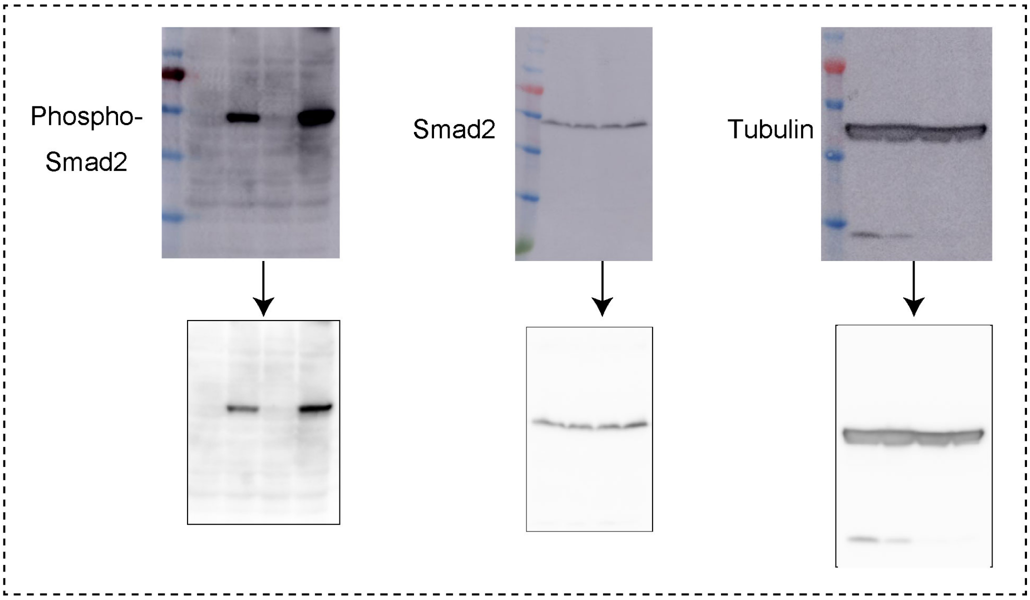

Supplemental Figure 3

Full blots for Figure 6a

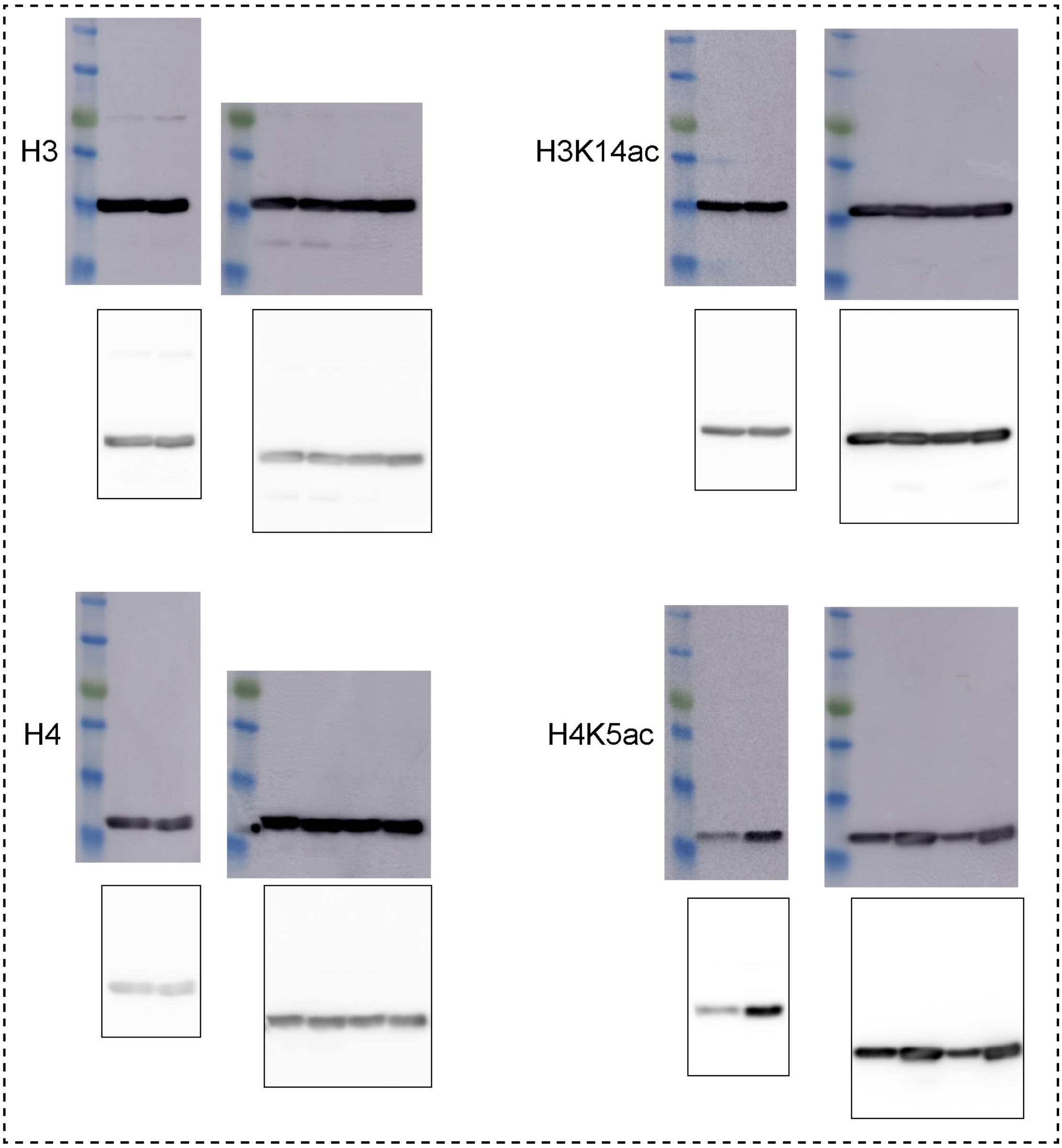

Supplement: Supplementary file 1 — Supplemental Figures [file 41598_2018_33881_MOESM1_ESM.pdf]
